# Intramuscular delivery of neural crest stem cell spheroids enhances neuromuscular regeneration after denervation injury

**DOI:** 10.1186/s13287-022-02877-1

**Published:** 2022-05-16

**Authors:** LeeAnn K. Li, Wen-Chin Huang, Yuan-Yu Hsueh, Ken Yamauchi, Natalie Olivares, Raul Davila, Jun Fang, Xili Ding, Weikang Zhao, Jennifer Soto, Mahdi Hasani, Bennett Novitch, Song Li

**Affiliations:** 1 Departments of Bioengineering and Department of Medicine, University of California, Los Angeles, USA; 2 David Geffen School of Medicine, University of California, Los Angeles, USA; 3 Division of Plastic and Reconstructive Surgery, Department of Surgery, National Cheng Kung University Hospital, College of Medicine, National Cheng Kung University, Tainan, Taiwan; 4 Department of Neurobiology, University of California, Los Angeles, USA

**Keywords:** Neural crest stem cell, Spheroid, Neuromuscular junction, Tissue engineering, Peripheral nerve injury, Regenerative medicine, Organ-on-a-chip

## Abstract

**Background:**

Muscle denervation from trauma and motor neuron disease causes disabling morbidities. A limiting step in functional recovery is the regeneration of neuromuscular junctions (NMJs) for reinnervation. Stem cells have the potential to promote these regenerative processes, but current approaches have limited success, and the optimal types of stem cells remain to be determined. Neural crest stem cells (NCSCs), as the developmental precursors of the peripheral nervous system, are uniquely advantageous, but the role of NCSCs in neuromuscular regeneration is not clear. Furthermore, a cell delivery approach that can maintain NCSC survival upon transplantation is critical.

**Methods:**

We established a streamlined protocol to derive, isolate, and characterize functional p75^+^ NCSCs from human iPSCs without genome integration of reprogramming factors. To enhance survival rate upon delivery in vivo, NCSCs were centrifuged in microwell plates to form spheroids of desirable size by controlling suspension cell density. Human bone marrow mesenchymal stem cells (MSCs) were also studied for comparison. NCSC or MSC spheroids were injected into the gastrocnemius muscle with denervation injury, and the effects on NMJ formation and functional recovery were investigated. The spheroids were also co-cultured with engineered neuromuscular tissue to assess effects on NMJ formation in vitro*.*

**Results:**

NCSCs cultured in spheroids displayed enhanced secretion of soluble factors involved in neuromuscular regeneration. Intramuscular transplantation of spheroids enabled long-term survival and retention of NCSCs, in contrast to the transplantation of single-cell suspensions. Furthermore, NCSC spheroids significantly improved functional recovery after four weeks as shown by gait analysis, electrophysiology, and the rate of NMJ innervation. MSC spheroids, on the other hand, had insignificant effect. In vitro co-culture of NCSC or MSC spheroids with engineered myotubes and motor neurons further evidenced improved innervated NMJ formation with NCSC spheroids.

**Conclusions:**

We demonstrate that stem cell type is critical for neuromuscular regeneration and that NCSCs have a distinct advantage and therapeutic potential to promote reinnervation following peripheral nerve injury. Biophysical effects of spheroidal culture, in particular, enable long-term NCSC survival following in vivo delivery. Furthermore, synthetic neuromuscular tissue, or “tissues-on-a-chip,” may offer a platform to evaluate stem cells for neuromuscular regeneration.

**Supplementary Information:**

The online version contains supplementary material available at 10.1186/s13287-022-02877-1.

## Introduction

Muscle denervation has broad etiologies, occurring in trauma and motor neuron diseases such as peripheral nerve injury, amyotrophic lateral sclerosis, spinal muscular atrophy, Guillain–Barré syndrome, and Charcot–Marie–Tooth disease, as well as neuropathies of diabetes and alcoholism, degenerative disk disease, pernicious anemia, and intensive care unit-acquired weakness. Peripheral nerve (PN) injury alone affects over one million people worldwide each year. The resultant motor impact can contribute to consequences ranging from weakness or loss of functional independence, to respiratory failure and mortality, depending on the nerve(s) involved [1–3].

Despite the prevalence and severe implications of muscle denervation, there is currently no effective therapy to regenerate nerve injuries beyond a critical length (1 cm [cm] in rodents and 3 cm in humans), and much of the underlying pathophysiology remains unclear [1, 2]. Prognosis varies widely depending on nature of injury or illness, delay before intervention, and patient characteristics [3, 4]. Surgical methods include nerve transection repair with end-to-end anastomosis; if such primary repair is not possible, nerve grafts (whether autografts or from other sources [5–8]), nerve conduits, nerve and nerve-muscle pedicles transfer may be considered. In the event of lack of a distal nerve segment for anastomosis, direct nerve transplantation (neurotization) into the muscle may be performed. However, these have led to only partial recovery of function at best [6, 9, 10], suggesting that although acceleration of axon growth is critical for nerve regeneration, attention beyond the nerve to its connections is another major barrier of functional recovery—specifically, the reinnervation and reformation of neuromuscular junctions (NMJs) [3, 4, 11, 12].

Regeneration of NMJs is thought to be supported by growth-promoting activity and signaling from the injured nerve, Schwann cells, and target muscle [3, 13, 14]. Nevertheless, a sustained release of growth factors (GFs), whether via exogenous delivery or endogenously overexpressed genes for neurotrophic factors, results in variable to limited improvement [6, 15, 16]. Until optimal release doses and kinetics are identified, genetic engineering tools such as CRISPR/Cas9, used effectively for treating genetic diseases like Duchenne muscular dystrophy [17, 18], remain of limited benefit here. Stem cell transplantation has advantages over synthetic manipulation of these complex and incompletely understood paracrine programs, as transplanted cells not only are capable of acting as environmentally responsive reservoirs of physiologic levels of paracrine signals, but also offer additional benefits such as cell communication, migration, and differentiation [9]. Previous cellular therapies for denervation have more often focused on the damaged nerve itself and have incompletely addressed the unmet needs for NMJ regeneration [3, 19]. In addition, effective types and sources of stem cells for NMJ regeneration have not been identified.

Neural crest stem cells (NCSCs) are stem cells that can be isolated and differentiated from embryonic stem cells (ESCs), embryonic neural crest, and induced pluripotent stem cells (iPSCs) and are found in low abundance in adult tissues. They have the capacity to differentiate into cell types of all three germ layers, in particular cells in the peripheral nervous system like peripheral neurons and Schwann cells [20–22]. Transplantation of NCSCs into nerve conduits that bridge transected nerve promotes nerve regeneration and functional recovery through Schwann cell differentiation and trophic signaling [23]. During muscle development, NCSCs play critical signaling roles in regulating early and sustainable myogenesis as well as regulating maintenance and differentiation of the skeletal muscle progenitor pool [24, 25]. NCSCs thus represent a developmentally relevant cell type for nerve-muscle regeneration. In juxtaposition, human bone marrow (BM)-derived mesenchymal stem cells (MSCs) are multipotent adult stem cells that have been shown to generate BM stroma (including adipocytes and local functional organization of new blood vessels) that support hematopoiesis [26–28], along with immunomodulatory roles [27]. Their implantation in nerve conduits has also improved nerve regeneration and is thought to be instead due to neovascularization and modulation of the influx of inflammatory milieu at nerve injury sites [29]. For intramuscular transplantation of BM-MSCs (hereafter referred to simply as MSCs unless otherwise specified) or NCSCs, however, effects for PNI were unknown.

After in vivo transplantation, major barriers exist for stem cell therapies regardless of cell type or body system in the form of low retention and survival rate [30, 31]. Over 95% of cells typically migrate out of the target site within 24–48 h, and of those remaining about 99% die by 4–6 weeks, leaving just 0.05% of the original delivery to exert effects [32]. Besides co-injecting biomaterials [8], aggregate “spheroidal” culture in some cell types enhances cell viability, helps preserve phenotype and function of stem cells, and increases protein synthesis [33–36]. MSCs have also been effectively made into spheroids with therapeutic benefit [37–40]. Following this rationale, we sought to investigate whether spheroids of NCSCs and MSCs could promote NMJ formation and neuromuscular functional recovery. Furthermore, just as recent development of synthetic neuromuscular tissues and organ-on-a-chip systems has demonstrated promising results in disease modeling and drug screening [41–45], we used such a system to examine spheroids with an in vitro synthetic neuromuscular tissue (SyNMT) model to demonstrate the feasibility of using SyNMT to screen stem cells for neuromuscular regeneration.

## Materials and methods

### iPSC culture and NCSC derivation

We used two sources of iPSCs to test different NCSC lines. The first was an iPSC line generated from human skin fibroblasts (Thermo Fisher, C0135C) without the integration of reprogramming factors into the genome, as previously described [23] (Additional file 1: Fig. S6A). The second was a line of human iPSCs from a collaborator (Joseph Wu, Stanford University). To derive NCSCs, iPSCs were grown as embryoid body (EB)-like floating cell aggregates in suspension culture for 6 days in serum-free NCSC induction medium consisting of KnockOut DMEM/F12 (Gibco), StemPro neural supplement (Invitrogen), 20 ng/ml basic fibroblast growth factor (bFGF; PeproTech, 100-18B), and 20 ng/ml epidermal growth factor (EGF; PeproTech, AF-100-15). EBs were then allowed to adhere to Matrigel-coated dishes and dissociated and replated after rosette formation. NCSCs were purified by magnetic-activated cell sorting (MACS, Miltenyi Biotec) for p75 (Miltenyi Biotec, #130-097-127) positivity and SSEA-4 negativity (Miltenyi Biotec, #130-097-855), twice each. ROCK inhibitor Y27632 2HCL (Fisher, 50-863-7) was used with passaging. Differentiation assays were performed as previously [23]. For expression of luciferase, cells were transduced with EF1α-Fluc2-PGK-Puro lentiviral vector (UCLA Vector Core) in OptiMEM media (Gibco; 31985062) with protamine sulfate (1:600) for 24 h, followed by expansion in normal NCSC media for another 2 days prior to a week of puromycin selection. Cells were used or frozen thereafter.

### NCSC spheroid characterization

Spheroid formation was scaled up via centrifugation method in microwell plates (AggreWell™), with size control via seeding cell suspension density. Survival was assessed by live–dead stain (Invitrogen, R37601), and the in vitro secretome after 5 days was assessed with a commercially available assay for 40 common GFs (Quantibody Assay 1, RayBiotech) in the conditioned medium (CdM). Custom-written MATLAB (MathWorks) code was developed to analyze and interpret assay densitometry calibrated to a standard curve. Normalization was performed by reporting the relative level with respect to the GF with the lowest concentration.

### MSC culture

Human MSCs were obtained without identifying patient information from the Texas A&M University Health Science Center College of Medicine, which follows the Tulane Center for Gene Therapy protocol for cell isolation. In short, they isolated nucleated cells from bone marrow aspirates of the iliac crest of normal, healthy adult volunteers by Ficoll/Paque density gradient, resuspended in CM (alpha-MEM, 20% FBS, P/S), and cells adherent after 24 h were collected as “MSCs” [46] for distribution. Cells were not used beyond passage 6 for our experiments for consistent phenotype [47].

### Poly(dimethylsiloxane) (PDMS) microgroove fabrication

Microgrooves were fabricated [48, 49] using silicon wafers from Sylgard 184 Silicone Elastomer with groove dimensions 20 µm width (W) × 3 µm height (H) chosen for this particular system based on preliminary data using 10–40 µm W × 0-12 µm H (data not shown) (Additional file 1: Fig. S2). Storage modulus, as assessed with rheometry of samples, was adjusted by tuning the proportion of Elastomer base and cross-linker (37:1) and baking conditions (30 min at 125 °C). A Discovery Hybrid Rheometer (TA Instruments HR-2) fitted with a 9-mm (mm) parallel-plate geometry and crosshatched base plate were used to interrogate the storage modulus at 37 °C using a 0.1–1.0 Hz frequency sweep with a 1% strain rate. The average of at least three samples is reported.

Microgrooves were cut to size for placement in 24-well dishes and then sonicated in 70% ethanol for 15 min for sterilization, followed by several phosphate-buffered saline (PBS) washes before air-drying. Chips were sterilely placed in 24-well dishes groove-side up with forceps. Plasma treatment was followed by overnight hESC-qualified Matrigel (Corning; #354277) coating before cell seeding.

### Electrospinning nanofibers

Nanofiber scaffolds were electrospun from a poly-l-lactide acid (PLLA) solution as previously described [49]. Briefly, fibers were aligned by uniaxially stretching the electrospun membrane by 200% deformation in length in a 60 °C water bath [49]. Stretched fibers were allowed to dry before cutting to size and mounting into 24-well dishes on double-stick tape. Scaffolds were sterilized in their wells with 70% ethanol for 15 min followed by several PBS washes before air-drying. Plasma treatment was followed by overnight Matrigel coating before cell seeding.

Nerve conduits were electrospun hollow tubes composed of 2:1 poly(lactide-co-caprolactone)/PLLA, 10 mm in length with a 2 mm inner diameter, as described in our previous publications [23, 50].

### SyNMT assembly and culture

Rodent cells were chosen to constitute this synthetic tissue model due to our rodent in vivo model. Mouse C2C12s or chemically induced myogenic cells (ciMCs) were seeded onto Matrigel-coated microgrooves or nanofibers in expansion medium consisting of Dulbecco’s Modified Eagle Medium (DMEM; Gibco, 11965), 10% fetal bovine serum (FBS; Gibco, 26140079), and 1% penicillin/streptomycin (P/S; GIBCO, 15140122), with chemicals (20 μM forskolin, 20 μM RepSox, 50 μg/ml ascorbic acid [Sigma], and 50 ng/ml bFGF [Stemgent Inc.]) in the case of ciMCs [51]. The media was changed once every 2–3 days. Two days after seeding, C2C12s were switched over to low-serum media (DMEM, 2% horse serum media [HSM], 1% P/S) to facilitate fusion and differentiation into myotubes.

Mouse ESC-derived GFP^+^-MNs were prepared as described [52, 53], with smoothened agonist (SAG, 1uM) instead of purmorphamine and maintenance media of the core serum-free MN media with 1 × N2, and 10 ng/ml each of BDNF, GDNF, and CNTF. MNs were seeded onto myotubes after 5 days. If stem cells were added, the spheroids were seeded within the next day after MN adhesion either with the SyNMT culture, or in Matrigel-coated 0.4-µm Transwells (Falcon, #353095). Out of necessity for myotube survival, for 4 days total before immunohistochemical analysis, myotube and MN co-cultures used media comprised of 1:1 MN media:HSM media.

For NMJ quantification, Z-stacks were taken in representative fields of at least three different MN neurospheres per condition at 40 × magnification (see “Immunofluorescent staining and microscopy” section). Maximum intensity projections were created from the Z stacks using the Zeiss software, and the areas of innervation were determined to be locations where axons stained by neurofilament-M (NF-M) entered α-Bungarotoxin (α-Btx)-stained NMJs.

### Rat care and surgery

Full-time vivarium staff and care was provided at the animal facility at the University of California, Los Angeles (UCLA). The vivarium was approved by the AACLA, and the animal studies were approved by the IACUC of the institution. Adult female athymic nude rats (NIH rnu, Charles River) were monitored and fed with standard chow by UCLA animal technicians daily, as well as monitored daily by UCLA veterinarians. All animals were additionally checked by us daily for normal behavior and signs of infections, distress, pain (e.g., autotomy), or weight loss. Any neuropathic pain and autotomy were treated with daily oral gabapentin and topical silver sulfadiazine as prescribed by UCLA’s veterinarians.

All experimental procedures with animals were approved by the UCLA ACUC committee and carried out according to the institutional guidelines. Adult female athymic nude rats (NIH rnu, Charles River) weighing 200–250 g and aged 2 months were used. Experimental animals in all control and cell groups were anesthetized by inhaled isoflurane and placed on a heat pad. The whole skin of the left hindlimb was shaved and disinfected with betadine and alcohol-soaked gauze three times each. The dorsal skin over the hip joint and gluteal muscle was cut and separated to expose the sciatic nerve. Sharp microsurgical scissors were used to remove 1 cm of the left sciatic nerve between the sciatic notch and the trifurcation of the sciatic nerve under surgical microscope. The nerve gap was bridged with a hollow electrospun tube fabricated as in the “Electrospinning nanofibers” section, using 8-0 nylon sutures on each side to anchor the tube to the connective tissue of the epineurium, as described in our previous publications.

Sampling of NCSCs for representative live–dead assay was used day-of surgery to assess viability. After nerve transection and bridging surgery, one million cells were resuspended prior to delivery in 50 μL sterile PBS, front-loaded into a 1-mL syringe, and then injected into the affected gastrocnemius muscle via insertion of a 19-gauge needle into the gastrocnemius muscle at the insertion point of the tibial branch of the sciatic nerve. The corresponding volume of saline without cells was injected instead for control animals with the same surgery performed. The gluteal muscle and skin wound were closed with 4-0 biodegradable and nylon sutures, respectively.

### In vivo imaging

Rats were anesthetized with isoflurane in a holding chamber, injected with luciferin (150 mg/kg, intraperitoneal), then moved to the optical scanner (IVIS Lumina II, Perkin Elmer) and after 7 min imaged dorsal-side up (10-min exposure) under maintenance anesthesia on isoflurane. Flux analysis was conducted with Living Image software (Caliper Life Sciences).

### Rat functional analysis

Functional recovery was assessed by gait video analysis to calculate sciatic function index (SFI), electrophysiological testing, and muscle wet weight, performed as previously [23]. SFI is defined here as = $-38.3*\frac{EPL-NPL}{NPL}+109.5*\frac{ETS-NTS}{NTS}+13.3*\frac{EIT-NIT}{NIT}-8.8$, where PL = print length = distance from heel to third toe, TS = toe spread = distance from first to fifth toe, ITS = intermediary TS = distance from second to fourth toe, N = normal, E = experimental [54]. Briefly, rats were videotaped in slow motion from below, walking across a transparent glass tunnel, and the paw print video stills were analyzed for appropriate print measurements for SFI.

PolyVIWE16 data acquisition software (Astro-Med, Inc.) was used to acquire data for electrophysiology analysis. Electrical stimuli (single-pulse shocks, 1 mA, 0.1 ms; using Grass Tech S88X Stimulator by Astro-Med Inc.) were applied under anesthesia (isoflurane, with heating pad at 37 °C) to the native sciatic nerve trunk at the point 1-mm proximal to the graft suturing point, and compound muscle action potentials (CMAPs) recorded in the gastrocnemius belly from 1 to 12 V or until a supramaximal CMAP was reached. Normal CMAPs from the un-operated contralateral side of sciatic nerve were recorded for comparison. The amplitude, response latency, and conduction velocity of the action potential were used to quantify the functional recovery of the regenerated peripheral nerve, with electrophysiological recovery rate defined as the ratio of the CMAP between the injured and contralateral normal hindlimb. Rats were thereafter euthanized by anesthetic overdose with secondary thoracotomy, and gastrocnemius muscles were then collected and wet weight recorded before flash-freezing OCT-coated tissues in liquid nitrogen-cooled isopentane and storing at − 80 °C for future histological analysis.

### Tissue histology and quantification

Gastrocnemius muscle was cryosectioned for hematoxylin and eosin (H&E) staining and immunostaining. Representative slices from throughout the gastrocnemius muscle were used for quantification and analysis. Muscle fiber area was quantified using ImageJ software via area tracing. Junction innervation was evaluated within these sections by identifying all *en face* NMJs as stained by αBtx that colocalized with axons as stained by NF-M (see antibodies and reagents below). Percent innervation was calculated by dividing the number of these junctions innervated with neurofilament by the total number of whole *en face* junctions, and normalized by animal to the innervation ratio of the uninjured side, calculated the same way.

### Immunofluorescent staining and microscopy

SyNMT samples were fixed in cold 4% paraformaldehyde (PFA; Electron Microscopy Sciences, #15710) for 15 min. For immunohistochemical analysis, washes were performed with 0.1% BSA (Miltenyi Biotec, #130-091-376) in PBS rather than PBS alone, which was used for all other non-SyNMT samples. Frozen tissue samples were fixed in ice-cold 4% PFA, rinsed with PBS, and permeabilized and blocked with 0.5% Triton X-100 (Sigma, T8787) in PBS with 5% normal donkey serum (NDS; Jackson ImmunoResearch, 017000121) for 30 min at room temperature (RT). Primary antibodies were diluted in blocking solution and applied overnight at 4 °C. After three 5-min PBS washes, secondary antibodies were applied diluted in 4% NDS, together with 4',6-diamino-2-phenylindole (DAPI; Invitrogen, D3571) for 1 hour at RT. Secondary antibodies conjugated to Alexa Fluor® 488 or Alexa Fluor® 546 (Life Tech, Thermo Fisher) were used. Samples were imaged with a Zeiss Axio Observer Z1 inverted fluorescence microscope (epifluorescence) or imaged with the Leica TCS-SP8-SMD inverted confocal microscope. Images were analyzed with ImageJ.

Primary antibodies used were: Sox10 (Cell Signaling, D5V9L), p75 (Abcam; Millipore MAB5386), HNK1 (Sigma, C6680), Tuj1 (Covance), S100β (Abcam, ab52642), αBtx (Invitrogen, 813422), GFP (Abcam, ab13970), MF-20 (mouse, DSHB), and/or STEM121 (Takara Bio, #Y40410), Fsp1 (Millipore, 07-2274), Pax7 (mouse, DSHB), MyoD (mouse, DSHB), MyoG (mouse, DSHB), laminin (Sigma, L9393), human nuclear antigen (HNA; Millipore, MAB1281), NF-M (ab9034, ab7794), GAP-43 (Novus, NBP1-41123SS), and smooth muscle α-actin (SMA; Abcam, ab5694).

Secondary antibodies used were from Life Tech (Thermo Fisher): donkey anti-mouse 488 (A21202) and 546 (A10036); donkey anti-rabbit 488 (A21206), 546 (A10040), and 647 (A31573); goat anti-chicken 488 (A-11039), and donkey anti-sheep 647 (A21448).

### Statistics

Data were reported as mean ± standard error of the mean (SEM). The sample size necessary to detect significant effect was estimated by using Power and Precision statistical software (Englewood, NJ) with the following information: minimum significant effect to be detected, data variation, power (0.8), and type I error rate (0.05). For two-sample comparison, two-tailed Student’s t test was used. For multiple-sample comparison, analysis of variance (ANOVA) was performed to detect whether a significant difference existed between groups with different treatments, and a multiple comparison procedure Bonferroni correction was used post-analysis to identify where the differences existed. A *p* value of 0.05 indicated significance, unless otherwise noted.

## Results

### Derivation and characterization of NCSCs and spheroids

**Fig. 1:**
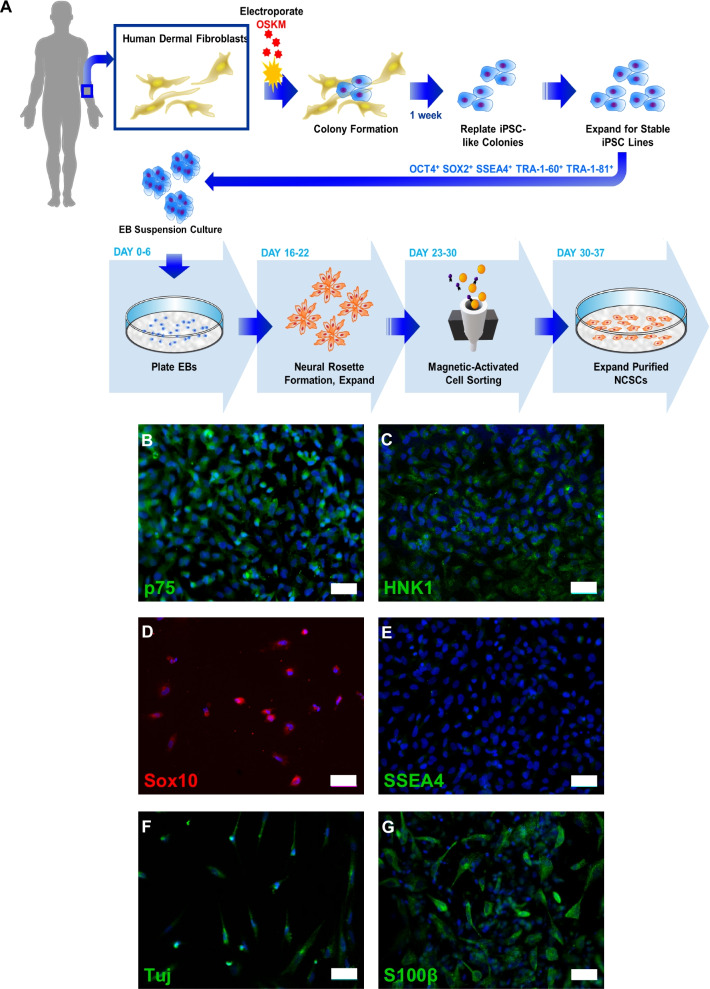
Human iPSC-derived NCSCs have multipotent potential. **A** iPSC and NCSC derivation process. **B**–**D** Expression of NCSC markers. **E** Expression of pluripotency marker SSEA4. Standard differentiation protocols were used to generate **F** peripheral neurons and **G** Schwann cells. Blue = DAPI, scale bar = 50 μm

We derived NCSCs from iPSCs following our previous protocol [23] (Fig. 1A). All NCSCs derived from different iPSC lines were expandable and homogeneously expressed NCSC markers p75 neurotrophin factor (p75), HNK1/N-CAM/CD57, and Sox10 (Fig. 1B–D) [55–57], with negativity for pluripotency markers such as SSEA4 (Fig. 1E). In addition, iPSC-NCSCs were multipotent and could differentiate into cell types of ectoderm (e.g., Schwann cells, peripheral neurons) (Fig. 1F–G) and mesoderm (chondrocytes, adipocytes, and osteoblasts) [50] lineage. Peripheral neurons were positive for neurofilament β-III tubulin (TUJ1) (Fig. 1F), and Schwann cells were positive for S100β (Fig. 1G).

**Fig. 2:**
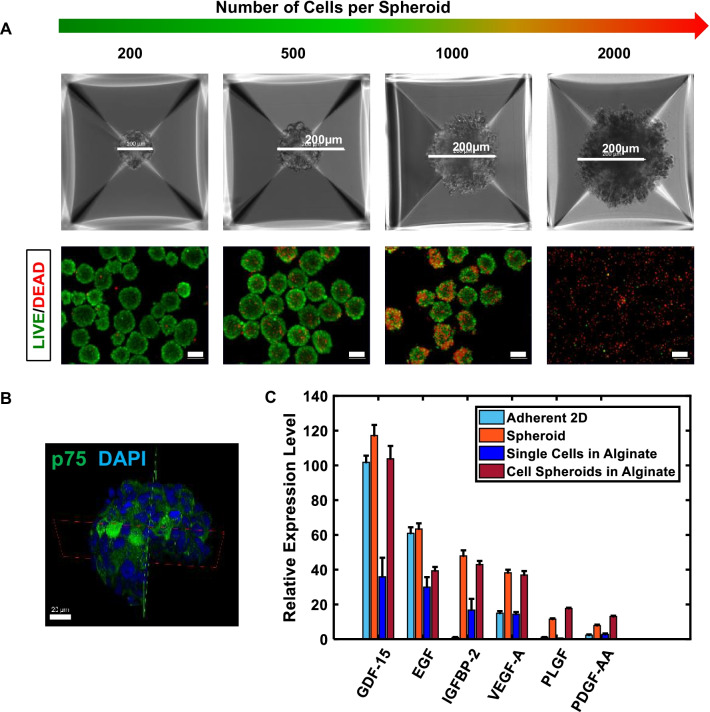
Human iPSC-derived NCSCs form spheroids with an enhanced regenerative secretome. **A** Size dependency of NCSC spheroid survival, Scale bar = 100 μm unless otherwise specified. **B** 3D quarter-cutaway of a spheroid stained for NCSC marker p75. Scale bar = 20 μm. **C** Regenerative secretome of NCSCs in various modalities. GF expression levels were reported relative to the GF with the lowest detected concentration, i.e., IGFBP-2 concentration in adherent 2D culture

Spheroidal culture in other cell types helps preserve phenotype and enhanced functionality, such as differentiation capacity and protein secretion [33, 34, 36]. Indeed, MSCs have also been made into spheroids, which improves cell survival and therapeutic benefit over single-cell suspension [37, 38] and monolayer [39, 40]. We first evaluated the impact, if any, of spheroid formation on NCSCs. Spheroid formation with diameters from 100 to 250 µm was studied in 50 µm increments, as controlled by varying cell seeding density (200–2000 cells/spheroid) into 1200-microwell plates. Live–dead staining showed that spheroids with 500 and fewer cells, a diameter approximately equal to the 150 µm diffusion limitation found in the literature [58, 59], had little cell death (Fig. 2A) and, furthermore, retained homogenous expression of p75 for 3 days after spheroid formation (Fig. 2B).

PN regeneration is thought to be supported by growth-promoting activity and signaling by the injured nerve, Schwann cells, and target muscle. Among secreted signals thought to be important are pro-regenerative growth factors acting for neurogenesis or axonal health and myogenesis (e.g., IGFBP-2 [60], VEGF [61]), among many others. Despite the higher rate of cell proliferation in two-dimensional culture, spheroidal culture (Fig. 2C; bars in orange and red) improved the secretion of such important GFs in neuromuscular function when compared with single-cell seeding (bars in blue) even with bulk encapsulation in hydrogel, a modality often of interest in cell therapies. Enhanced secretion of factors important in vascularization (IGFBP-2 [60], VEGF [61], PLGF [62], PDGF-AA [60]), immunomodulation (GDF-15 [63]), and maintenance of stem cell precursor proliferation (EGF [64, 65]) were also observed (Fig. 2C).

### NCSC spheroids drastically improved cell survival in vivo

**Fig. 3:**
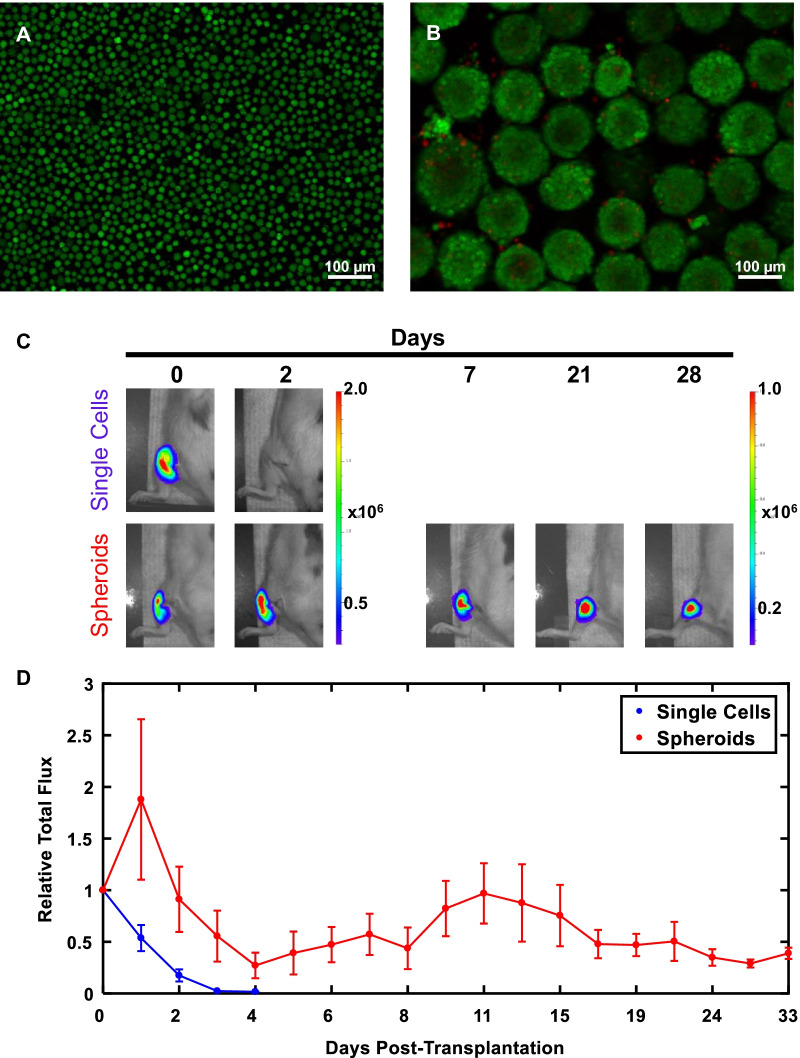
NCSC spheroids improve in vivo survival following transplantation. **A**, **B** NCSCs were subject to live–dead stain following ejection through the needle prior to transplantation. Green = live, Red = dead. **A** Single-cell suspension and **B** spheroids are shown. Scale bar = 100 µm. **C** Bioluminescent noninvasive tracking of luciferase-labeled NCSC survival (total flux, in p/s) after transplantation as single-cell suspension versus spheroids, n = 3 each. **D** Plot of total flux relative to original baseline directly following transplantation

We investigated whether spheroidal culture of NCSCs had any impact on the major clinical barriers of cell survival and retention relative to classical transplantation of single-cell suspensions. Luciferase-expressing NCSCs were used for noninvasive cell fate tracking. Using a rat model of sciatic nerve injury, NCSCs were transplanted intramuscularly into the gastrocnemius at the insertion of the sciatic nerve as either spheroids or single-cell suspension. Although robust survival was seen with syringe injection of single-cell suspensions and also spheroid suspensions prior to transplantation (Fig. 3A, B), fate was starkly different following transplantation. Whereas NCSCs transplanted in single-cell suspensions rapidly died off within 3 days, spheroids continued to survive and express bioluminescent signal (Fig. 3C), with flux signal equilibrating after 2.5 weeks at approximately 31% of original levels (Fig. 3D). Indeed, spheroid-delivered cells survived throughout 9 weeks of the trial (31.5 ± 0.06% at 9 weeks), underscoring the enhancement of NCSC cell survival in vivo by spheroids.

### NCSC spheroids, but not MSCs, enhanced functional recovery following denervation injury

**Fig. 4:**
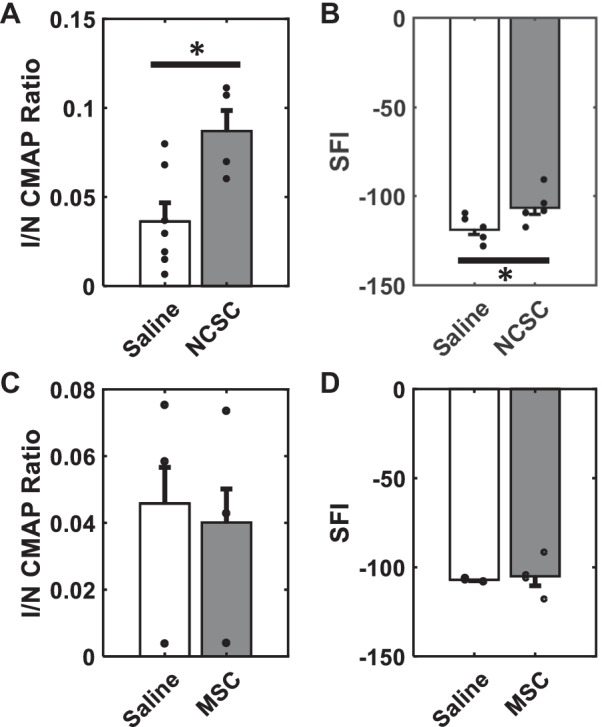
NCSCs but not MSCs improve functional recovery 4 weeks after stem cell transplantation. **A**, **B** Functional metrics for NCSC-transplanted rats, where I/N is the ratio of the injured (I) limb to normal (N) uninjured limb for each animal, CMAP = compound muscle action potential, SFI = sciatic functional index, controls were injected with saline rather than cells, *p < 0.05. **C**, **D** The same functional metrics, but for MSC-transplanted rats. n ≥ 3

BM-MSCs have been widely explored for regeneration, primarily for neurological, cardiovascular, and orthopedic indications, with an abundance of clinical trials [26–29, 66]. Given the reported utility of MSCs for regenerating a variety of tissues [67], we assessed both NCSCs and MSCs in vivo. Non-bioluminescent NCSCs and MSCs were formed into spheroids and transplanted into the same PN injury model and compared to sham (saline)-injected controls. Functional recovery was assessed at 4 weeks. In vivo neuromuscular functional communication was assessed by electromyographical analysis, a widely accepted method which records the electrical activity of muscle as it contracts in response to the motor neuron’s stimulating action potentials via needle electrodes inserted into the muscle of interest. Such electrophysiology revealed a significant 2.4-fold recovery of NCSC-injected versus control saline-injected animals (Fig. 4A, *p* < 0.05). Similarly, walking track analysis of gait revealed a significant improvement in NCSC- versus saline-injected animals using the sciatic functional index [54] (SFI, Fig. 4B,* p* < 0.05). In stark contrast with NCSC spheroids, and consistent with SyNMT predictions, all functional metrics following MSC transplantation were insignificantly different from controls (*p* > 0.7, Fig. 4C, D).

### NCSCs promoted NMJ formation in vivo

**Fig. 5:**
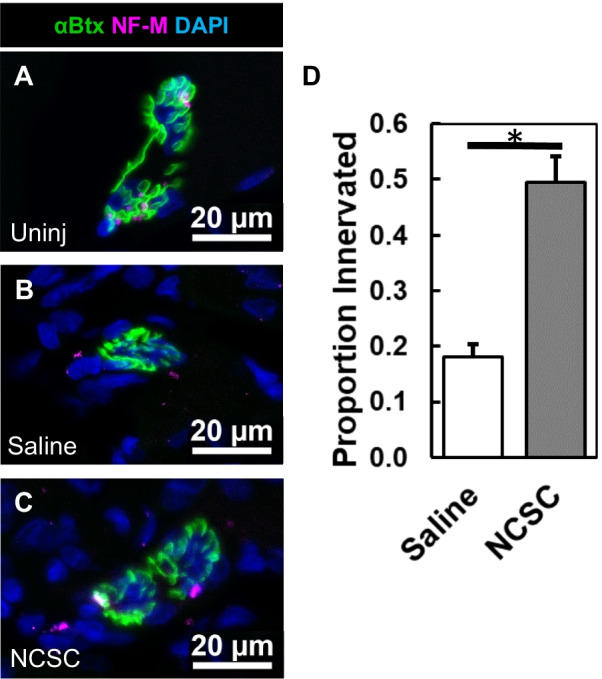
Neuromuscular histology is improved 4 weeks following NCSC transplantation. **A**–**C** Representative stains of NMJs, where uninj = uninjured limb, versus saline and NCSC injected injured limbs, n = 5 each. NF-M = neurofilament medium. **D** Quantification of NMJs innervated with NF-M relative to the total number of NMJs, normalized for each animal to the proportion innervated in its uninjured limb. *p < 0.05

We then examined the histological characteristics of regenerated muscle. Axons of pre-synaptic motor neurons were labeled with antibody for neurofilament-M (NF-M), while post-synaptic acetylcholine receptors were stained with αBtx; was deemed as innervation [68]. NCSC-treated animals (Fig. 5C) had 2.7-times higher ratios of innervated NMJs (*p* < 0.05, Fig. 5D) than saline controls (Fig. 5B) in the gastrocnemius on the injured side. The uninjured side's NMJ is shown for comparison (Fig. 5A). Sectioned muscle showed slight differences in muscle fiber area with NCSC injection compared to saline injection that did not reach statistical significance (Additional file 1: Fig. S1). Long-term axonal reinnervation was very apparent at 9 weeks (Additional file 1: Fig. S2). Staining for cell fate after 4 weeks revealed the transplanted NCSCs were neither S100β^+^ myelinating nor GAP43^+^ nonmyelinating Schwann cells, suggesting that NCSCs did not fully differentiate into Schwann cells in the muscle, and associated with various structures (Additional file 1: Fig. S3).

### In Vitro co-culture of NCSC spheroids, but not MSC spheroids, with SyNMT increased NMJ formation

To directly examine the capability of NCSC spheroids in promoting NMJ formation, we engineered an in vitro co-culture model by seeding myotubes [51] on aligned patterned scaffolds with ESC-derived motor neurons. Given the known importance of alignment for tissues with organized architecture like muscle [69–74], we fabricated micropatterned PDMS surfaces with parallel microgrooves (20 µm wide, which is wider than typical myoblast dimensions to enable cell spread and growth [49], and of physiological stiffness [75], storage modulus of 9.31 ± 0.21 kPa), or used electrospinning and then stretching technique to make aligned PLLA nanofiber scaffolds as described previously [48, 49] (Additional file 1: Fig. S4). On these scaffolds, C2C12 myotubes grew, aligned, and expressed MF-20, also known as sarcomeric myosin heavy chain (MHC), the fundamental unit of contractile function in adult muscle fibers. Within days of co-culture with constitutively GFP-expressing, ESC-derived motor neurons (MNs) [53], they formed structural NMJs, as defined by visualizing presynaptic GFP^+^ neural innervation of post-synaptic acetylcholine receptors positive for αBtx (Additional file 1: Fig. S4D, H). Interestingly, although both microgrooves and nanofibers aligned neuromuscular cells, the nanotopography of electrospun scaffolds was superior to microgrooves in growing myotubes with improved maturation. This was seen in the staining for adult sarcomeric MHC, in development of striations (Additional file 1: Fig. S4G inset), and in the 1.5-fold increase in myotube width relative to those on microgrooves (7.6 ± 0.5 µm on microgrooves, vs 11.4 ± 0.6 µm on nanofiber scaffolds, *p* < 0.05, Additional file 1: Fig. S4I). Electrospun scaffolds were thus used for stem cell therapy screening thereafter.

We then determined whether primary myogenic cells could successfully replace the myoblast cell line (C2C12) to create a SyNMT with increased relevance to normal physiology. To address this question, we turned to a source of primary myogenic cells known as chemically induced myogenic cells [51] (ciMCs; Additional file 1: Fig. S5), which were enriched by differential adhesion of mouse skin fibroblasts and expanded by using a cocktail containing forskolin and a TGF-β inhibitor. Co-culture of ciMCs with motor neurons on electrospun scaffolds also effectively aligned and formed NMJs (Additional file 1: Fig. S4J).

NCSC or MSC spheroids were added to SyNMT and co-cultured for four days because NMJ formation, which is the first critical step for neuromuscular regeneration, is known to occur by this time [76]. Consistent with in vivo findings, NCSC but not MSC spheroids increased NMJ formation (Additional file 1: Fig. S6). Conditioned media (CdM) of NCSCs in the Transwell co-culture of NCSC SyNMT was insufficient to recapitulate the apparent contact-dependent benefits (Additional file 1: Fig. S6). STEM121 staining of human cytoplasm suggested tendencies of NCSCs to associate and align with axons (Additional file 1: Fig. S7), perhaps related to the observed contact dependency of NCSC improvements.

## Discussion

Severe peripheral nerve injury (PNI) remains without effective therapy for functional recovery [1, 2]. Cellular therapies for denervation have primarily focused on the damaged nerve in the past, and the optimal types of stem cells to promote muscle innervation, critical for functional recovery [3, 77, 78], have not been well established [79–81]. Here, we highlighted the unique potential of delivery of NCSC spheroids into affected muscle for neuromuscular regeneration after denervation injury. This therapy that targeted both nerve and muscle and their connections (i.e., NMJs) was found to be more effective for functional recovery. We also demonstrated the role a synthetic tissue model could play in evaluating stem cell therapies ex vivo.

NCSCs have bioactivity with both nerves and muscle, which could underlie their unique positive effects on PN regeneration. NCSCs originate Schwann cells, which dedifferentiate after PNI to become the drivers of the remarkable regeneration of which peripheral nerves are capable [57, 82]. NCSCs are also critical signalers to muscle progenitor cells (MPCs) in early muscle formation, enabling balanced, sustainable, and progressive MPC differentiation for appropriate myogenesis [20–22, 24, 25]. NCSC derivatives such as sensory nerve and Schwann cells control arterial differentiation and patterning as well [83]. Pathways that facilitate myogenesis and vascularization during development (e.g., neuregulin, Delta1 signaling) may feasibly be recapitulated by NCSCs in maintenance or regeneration of neuromuscular function after nerve injury; in our PNI model, NCSC spheroids significantly improved in vivo gait and electrophysiologic functionality (Fig. 4). By definition, for stronger muscle electric contractile activity to be measured in vivo at the muscle (here, the gastrocnemius at the insertion of the sciatic nerve), recruitment of motor units must have been improved, and functional NMJs must therefore have been present to enable this transmission of electrical stimulation from the motor neuron (here, the sciatic nerve proximal to the conduit suturing point). This principle has made electrophysiological testing one of the strongest pieces of evidence for functional NMJs in a live subject that one can achieve, as seen by the broad use of electromyography (EMG) as a clinical diagnostic tool for diseases affecting the neuromuscular junction.

In contrast to the NCSCs, our particular human BM-MSCs (isolated CD146^+^ from iliac crest BM aspirates [46]; similar to long bones) are neither myogenic nor neurogenic in vivo*,* though they have been shown to generate structures supporting hematopoiesis and immune modulation [26–28]. Consistent with the concept of necessary nerve and muscle bioactivity for regeneration, MSCs did not enhance in vivo NMJ formation in comparison with saline-injected controls. This does not preclude the use of other MSC subtypes not evaluated here in potential utility for similar applications; MSCs from other sources are thought to have additional differentiation capabilities [26–28].

The etiology of NCSC benefit may stem in part too from the survival benefits of spheroid transplantation. Spheroidal culture can prevent anoikis-mediated death of single cells in suspension and enhances the survival of many cell types of varying maturity, particularly of the neural lineage [33–35], but had not previously been assessed for impact on NCSCs. Intramuscular transplantation of spheroids rather than conventional single-cell suspensions enabled robust long-term NCSC survival and engraftment for up to 9 weeks, which otherwise would have been limited to under four days (Fig. 3C, D). This dramatic improvement in NCSC survival is critical for the improvement in clinical outcome.

Beyond survival, spheroidal culture also affected secretion profiles of NCSCs in vitro*.* Compared to single-cell-plated conditions in flat 2D culture, NCSC spheroids secreted increased pro-regenerative growth factors acting for neurogenesis or axonal health and myogenesis, vascularization, immunomodulation, and maintenance of stem cell precursor proliferation (Fig. 2C). Moreover, NCSC spheroid co-culture in SyNMT increased NMJ formation and qualitative axonal density, but interestingly, in vitro NCSC spheroid conditioned media alone was insufficient to recapitulate these neuromuscular benefits (Additional file 1: Fig. S6). We also did not observe the differentiation of NCSCs into neural cells in our in vivo studies, but noticed their proximity to muscle, nerve, and microvessels (Additional file 1: Fig. S7). Thus, it is likely that cell–contact-dependent interactions with either cell adhesion-induced soluble factors or other cell adhesion-induced functional changes account for these pro-regenerative effects, rather than paracrine effects or differentiation-driven direct cell replacement. For instance, NCSCs may lend contact-mediated support to axons as known to occur with Schwann lineage cells and axons *in vivo* [57, 84].

The formation of NMJs is dependent on nerve terminal-derived signals, many contact-dependent, to underlying basal lamina and muscle and is additionally assisted by muscle-secreted factors [85]. Therefore, cells that mediate this interplay are uniquely poised to facilitate neuromuscular regeneration after PNI. In our PNI model, NCSC spheroids significantly improved the proportion of innervated NMJs (Fig. 5), which is the first critical step for neuromuscular regeneration. NCSCs, while distinct from post-injury de-differentiated Schwann cells, may offer sufficient similarities to assist the natural regenerative processes [86]. Whether NCSCs recapitulate developmental roles in promoting muscle progenitor expansion after adult injury, or even of Schwann cell (whether de-differentiated or not) signaling in similar ways to promote muscle progenitor expansion, is uncertain but of great interest, and could be explored with single-cell tracking and fate analysis in the future. Furthermore, synthetic multi-tissue systems incorporating biophysical cues like ours have great potential in facilitating optimization of stem cell therapy regenerative effects, teasing out likely mechanisms of improvement in finer detail, and also moving beyond purifying and characterizing stem cells in isolation to improved evaluation of stem cells for translation to reduce the risk of complications. To our knowledge, this was the first time a synthetic tissue platform has been used to evaluate neuromuscular stem cell therapies as an adjunct to in vivo studies and therapy. Future iterations of this proof-of-concept could create systems of enhanced sophistication and relevance such as incorporating perfusion/shear flow, 3D alignment, and bundle formation such as with encapsulating ECM hydrogel, incorporating optogenetic systems to more easily study functional connectivity [42], and electrical stimulation for NMJ maturation [84], the last of which could also be combined with cell therapy in vivo for improved neuromuscular regeneration [87, 88]. Sourcing MNs and/or skeletal muscle from patient tissue or iPSCs would additionally provide a more personalized model of disease, therapeutic response, and cell therapy.

## Conclusions

Neural crest stem cells (NCSCs) pose greater and more direct advantages in promoting functional neuromuscular regeneration after intramuscular transplantation for PNI, in contrast with the more nonspecific secondary effects of CD146^+^ BM-MSCs. Appropriate stem cell type is critical for neuromuscular regeneration. Moreover, the biophysical effects of spheroidal culture on NCSCs provide a distinct advantage for peripheral nerve injury therapy in both bioactivity and NCSC survival. We highlight that both nerve and muscle are key components of regeneration following peripheral nerve injury and that the regeneration of NMJs for reinnervation is an important limiting step in functional recovery. Synthetic neuromuscular tissues or “tissues-on-a-chip” may offer a platform to further explore and evaluate stem cells for neuromuscular regeneration.

## Supplementary Information

**Additional file 1.** Supplemental research data and information.

## Data Availability

The raw/processed data are available upon request.

## Abbreviations

NMJs: Neuromuscular junctions
SyNMT: Synthetic neuromuscular tissue
NCSC: Neural crest stem cell
MSCs: Bone marrow-derived mesenchymal stem cells
PN: Peripheral nerve
cm: Centimeter
GF: Growth factor
ESCs: Embryonic stem cells
iPSCs: Induced pluripotent stem cells
BM: Bone marrow
PDMS: Poly(dimethylsiloxane)
PLLA: Poly-l-lactide acid
PBS: Phosphate-buffered saline
αBtx: α-Bungarotoxin
SFI: Sciatic function index
CMAPs: Compound muscle action potentials
NF-M: Neurofilament-M
H&E: Hematoxylin and eosin
S.E.M.: Standard error of the mean
ANOVA: Analysis of variance
MF-20: Also known as sarcomeric myosin heavy chain [MHC]
CdM: Conditioned media
