# Supplementary material for: Intramuscular delivery of neural crest stem cell spheroids enhances neuromuscular regeneration after denervation injury
Source: Stem Cell Res Ther. 2022 May 16;13:205. doi: 10.1186/s13287-022-02877-1 (PMC9109326; doi:10.1186/s13287-022-02877-1)
Supplement: Supplementary file 1 — Additional file 1. Supplemental research data and information. [file 13287_2022_2877_MOESM1_ESM.pptx]

## Slide 1
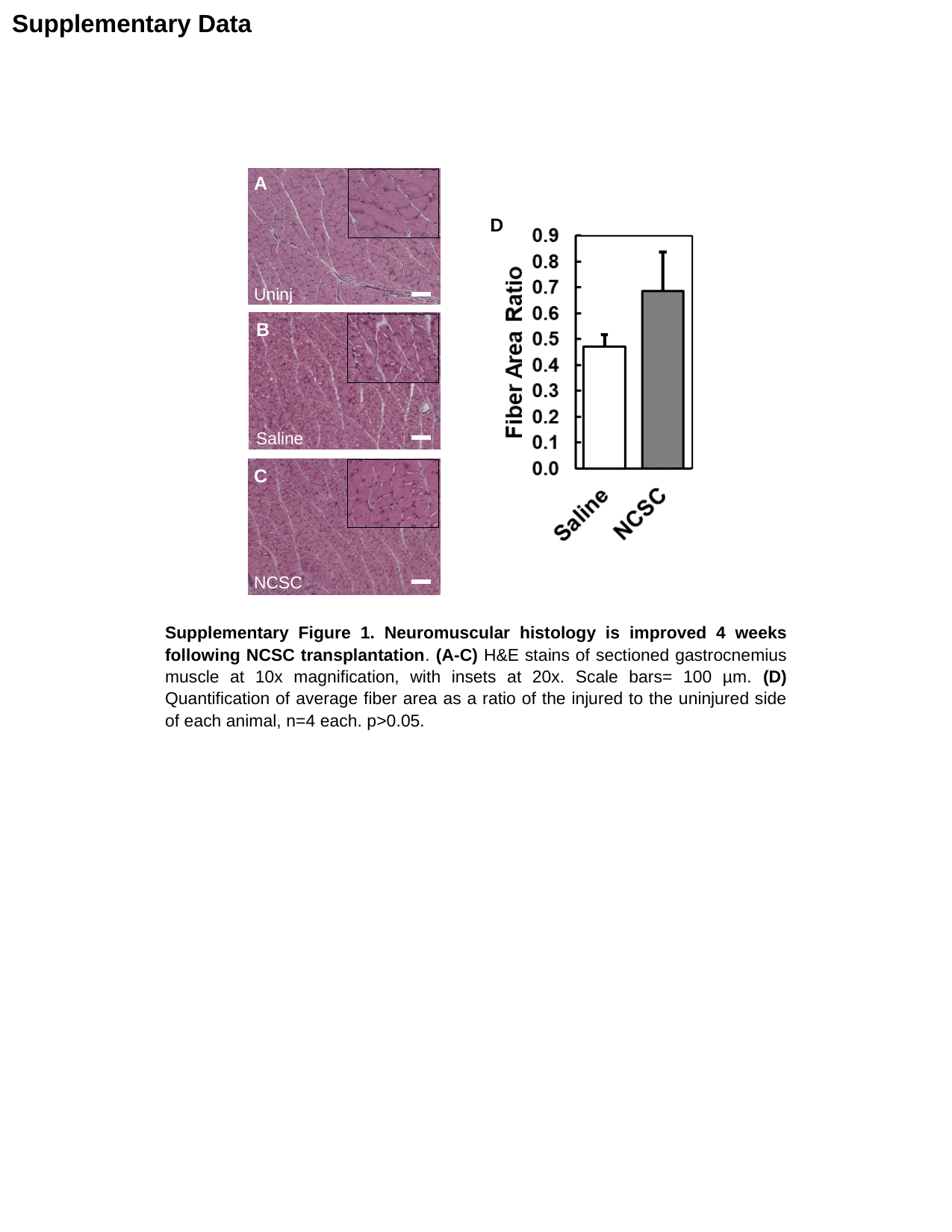

Supplementary Data
A
D
Uninj
B
Saline
C
NCSC
Supplementary Figure 1. Neuromuscular histology is improved 4 weeks following NCSC transplantation. (A-C) H&E stains of sectioned gastrocnemius muscle at 10x magnification, with insets at 20x. Scale bars= 100 µm. (D) Quantification of average fiber area as a ratio of the injured to the uninjured side of each animal, n=4 each. p>0.05.

## Slide 2
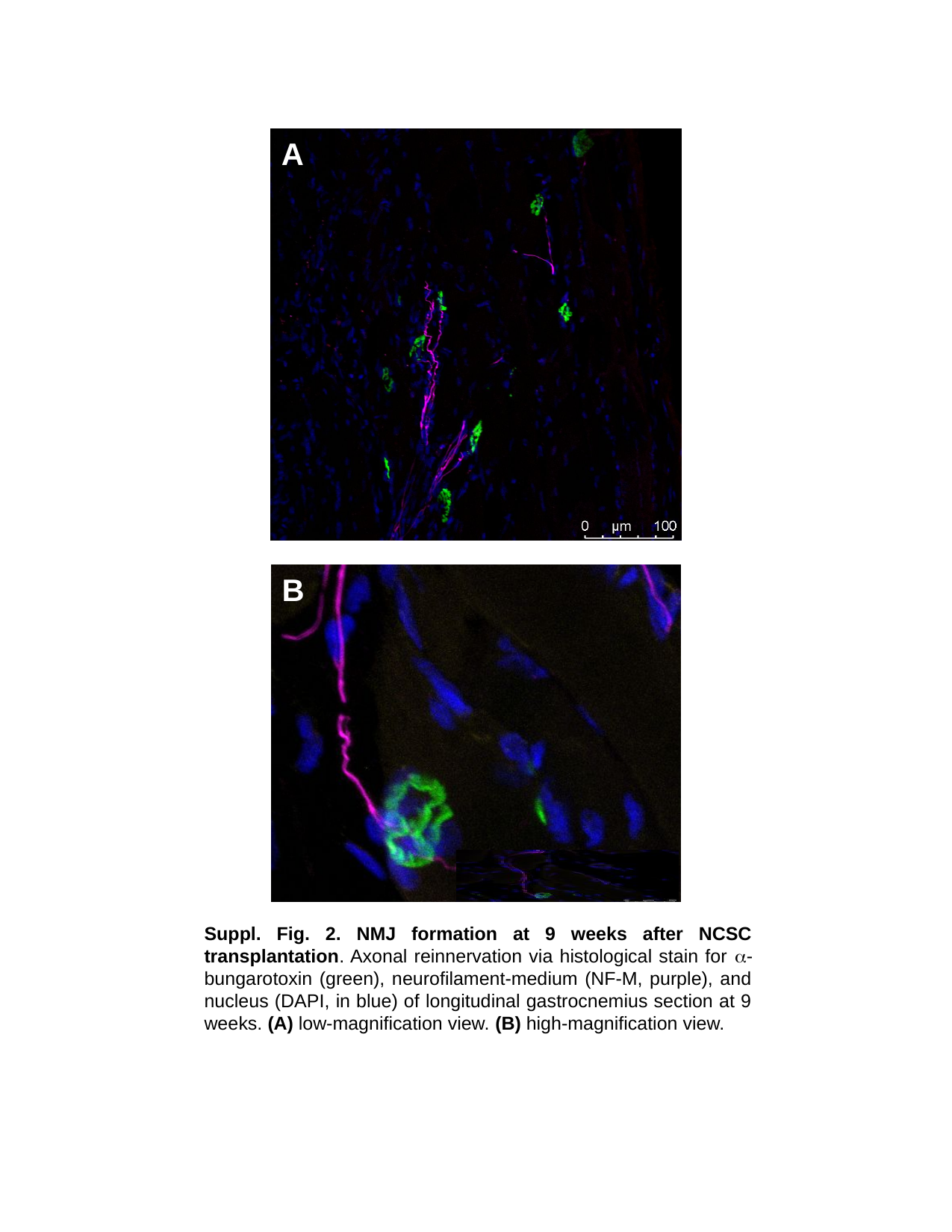

A
B
Suppl. Fig. 2. NMJ formation at 9 weeks after NCSC transplantation. Axonal reinnervation via histological stain for a-bungarotoxin (green), neurofilament-medium (NF-M, purple), and nucleus (DAPI, in blue) of longitudinal gastrocnemius section at 9 weeks. (A) low-magnification view. (B) high-magnification view.

## Slide 3
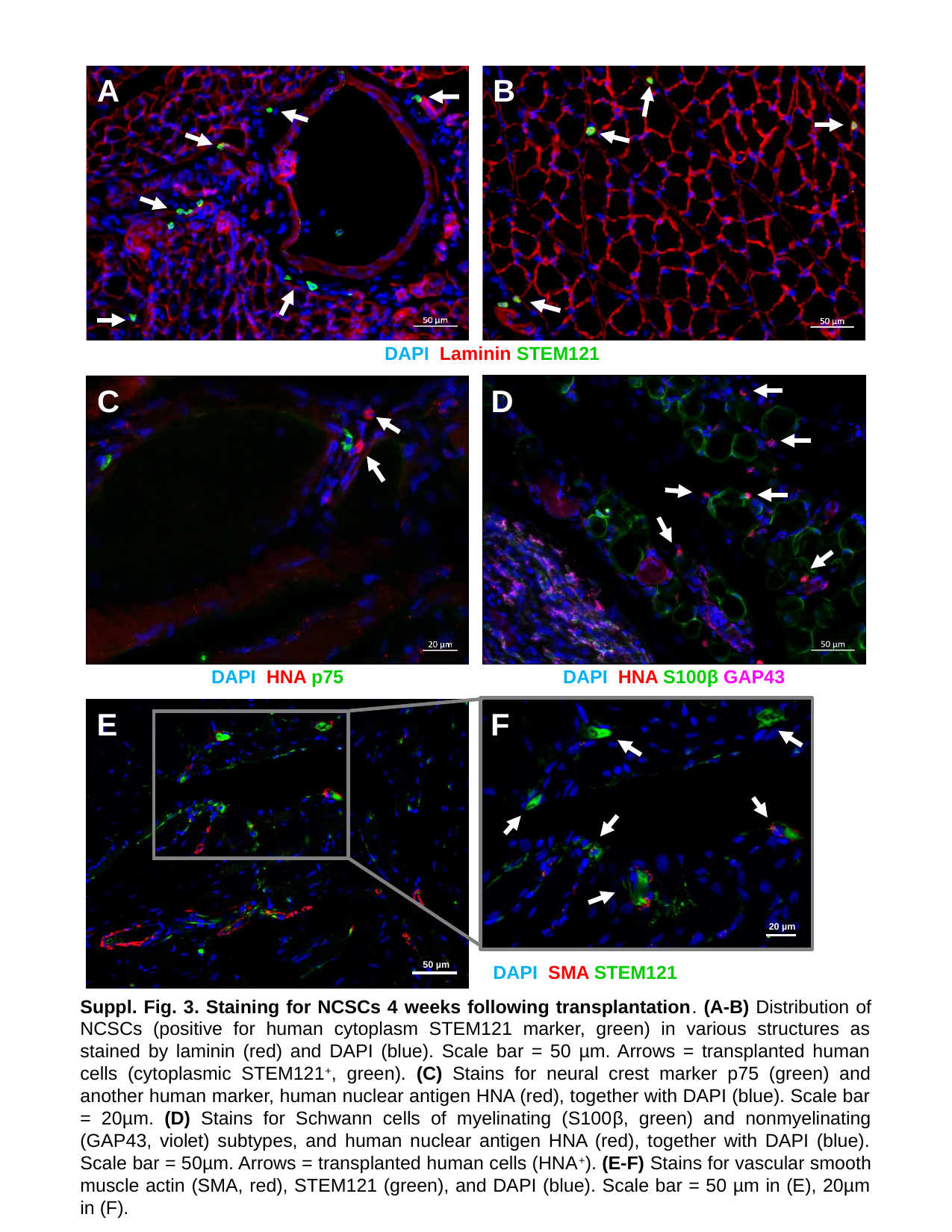

A
B
DAPI Laminin STEM121
C
D
DAPI HNA p75
DAPI HNA S100β GAP43
E
F
20 µm
50 µm
DAPI SMA STEM121
Suppl. Fig. 3. Staining for NCSCs 4 weeks following transplantation. (A-B) Distribution of NCSCs (positive for human cytoplasm STEM121 marker, green) in various structures as stained by laminin (red) and DAPI (blue). Scale bar = 50 µm. Arrows = transplanted human cells (cytoplasmic STEM121+, green). (C) Stains for neural crest marker p75 (green) and another human marker, human nuclear antigen HNA (red), together with DAPI (blue). Scale bar = 20µm. (D) Stains for Schwann cells of myelinating (S100β, green) and nonmyelinating (GAP43, violet) subtypes, and human nuclear antigen HNA (red), together with DAPI (blue). Scale bar = 50µm. Arrows = transplanted human cells (HNA+). (E-F) Stains for vascular smooth muscle actin (SMA, red), STEM121 (green), and DAPI (blue). Scale bar = 50 µm in (E), 20µm in (F).

## Slide 4
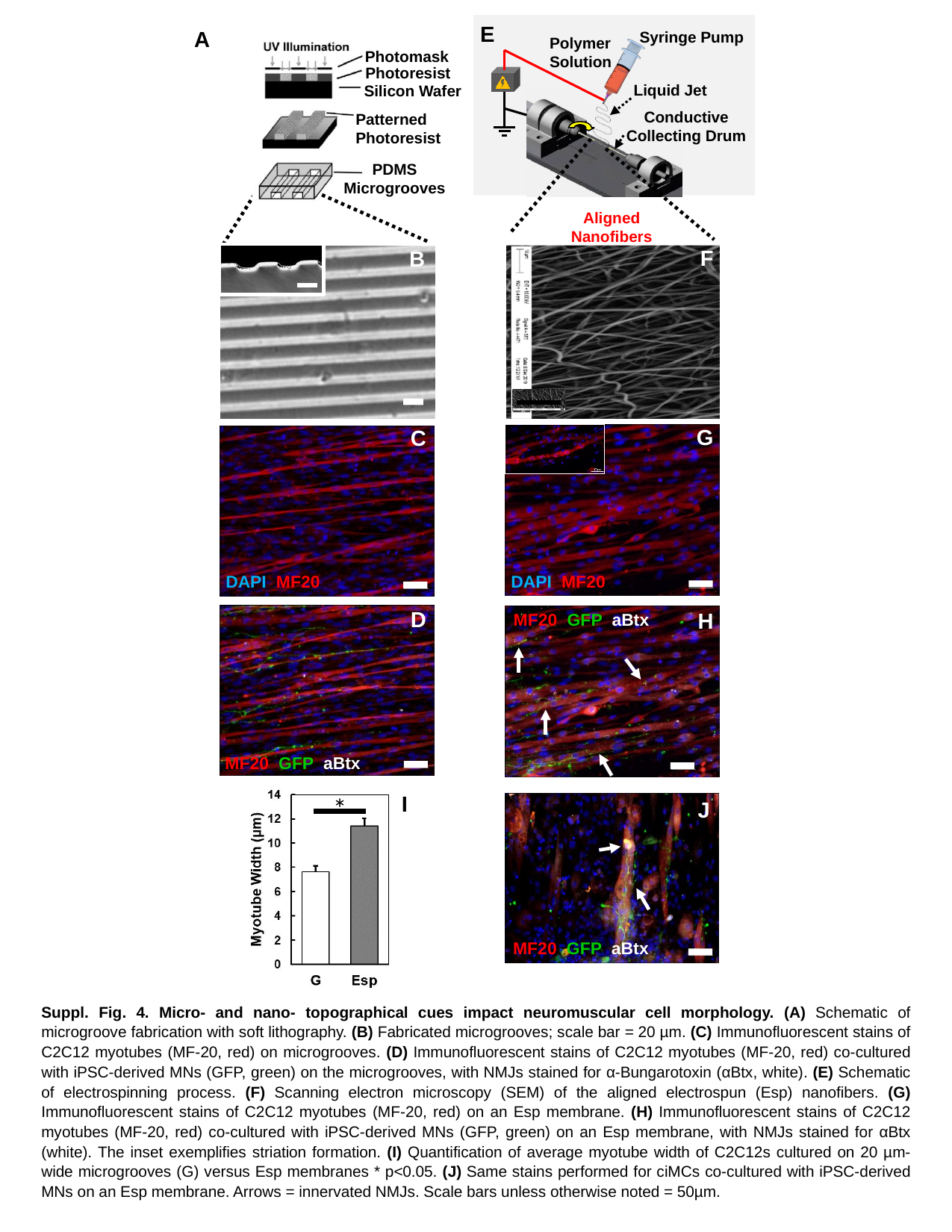

E
A
Photomask
Photoresist
Silicon Wafer
Patterned Photoresist
PDMS
Microgrooves
Syringe Pump
Polymer Solution
Liquid Jet
Conductive Collecting Drum
Aligned
Nanofibers
F
B
G
DAPI MF20
C
DAPI MF20
D
MF20 GFP aBtx
H
MF20 GFP aBtx
I
*
J
MF20 GFP aBtx
Suppl. Fig. 4. Micro- and nano- topographical cues impact neuromuscular cell morphology. (A) Schematic of microgroove fabrication with soft lithography. (B) Fabricated microgrooves; scale bar = 20 µm. (C) Immunofluorescent stains of C2C12 myotubes (MF-20, red) on microgrooves. (D) Immunofluorescent stains of C2C12 myotubes (MF-20, red) co-cultured with iPSC-derived MNs (GFP, green) on the microgrooves, with NMJs stained for α-Bungarotoxin (αBtx, white). (E) Schematic of electrospinning process. (F) Scanning electron microscopy (SEM) of the aligned electrospun (Esp) nanofibers. (G) Immunofluorescent stains of C2C12 myotubes (MF-20, red) on an Esp membrane. (H) Immunofluorescent stains of C2C12 myotubes (MF-20, red) co-cultured with iPSC-derived MNs (GFP, green) on an Esp membrane, with NMJs stained for αBtx (white). The inset exemplifies striation formation. (I) Quantification of average myotube width of C2C12s cultured on 20 µm-wide microgrooves (G) versus Esp membranes * p<0.05. (J) Same stains performed for ciMCs co-cultured with iPSC-derived MNs on an Esp membrane. Arrows = innervated NMJs. Scale bars unless otherwise noted = 50µm.

## Slide 5
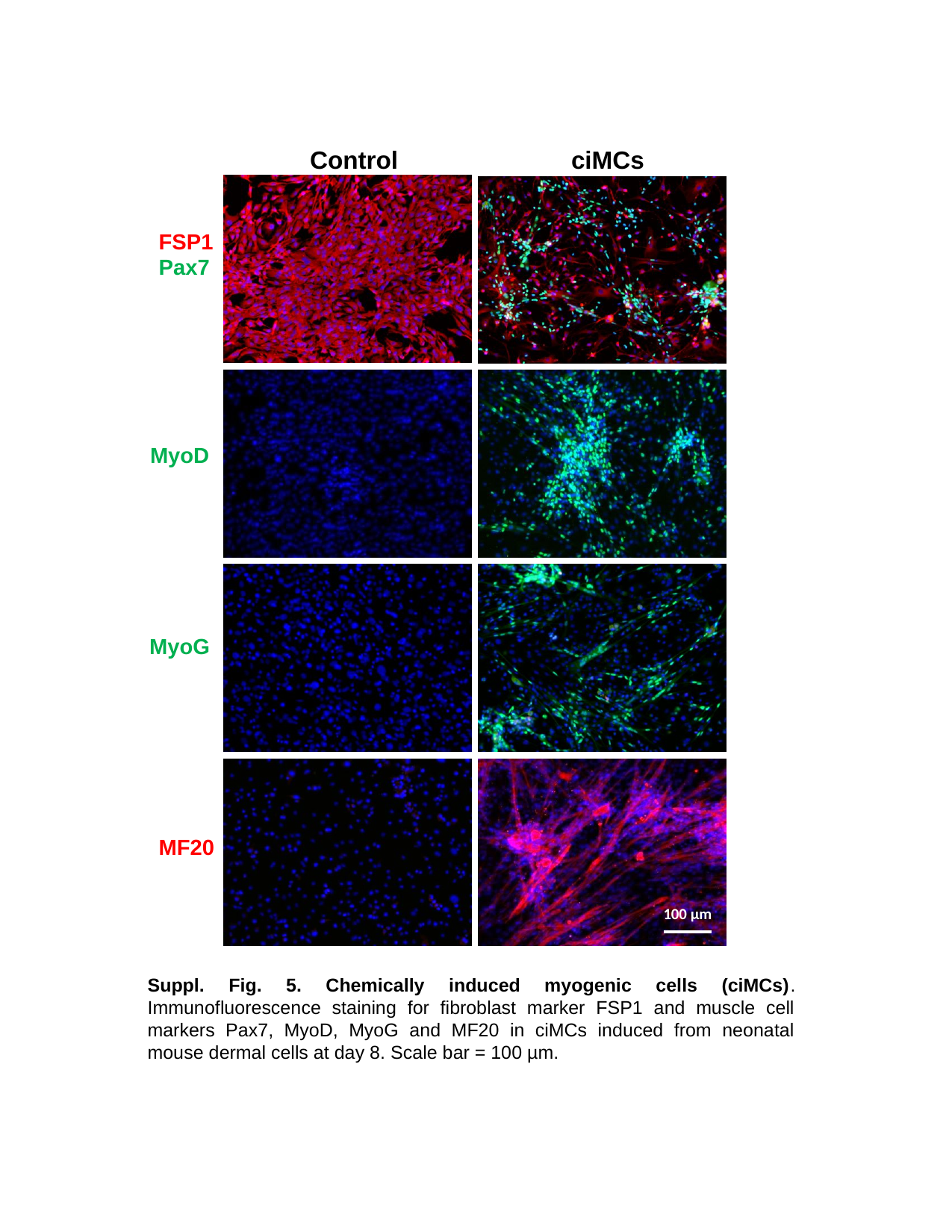

Control
ciMCs
FSP1
Pax7
MyoD
MyoG
MF20
100 µm
Suppl. Fig. 5. Chemically induced myogenic cells (ciMCs). Immunofluorescence staining for fibroblast marker FSP1 and muscle cell markers Pax7, MyoD, MyoG and MF20 in ciMCs induced from neonatal mouse dermal cells at day 8. Scale bar = 100 µm.

## Slide 6
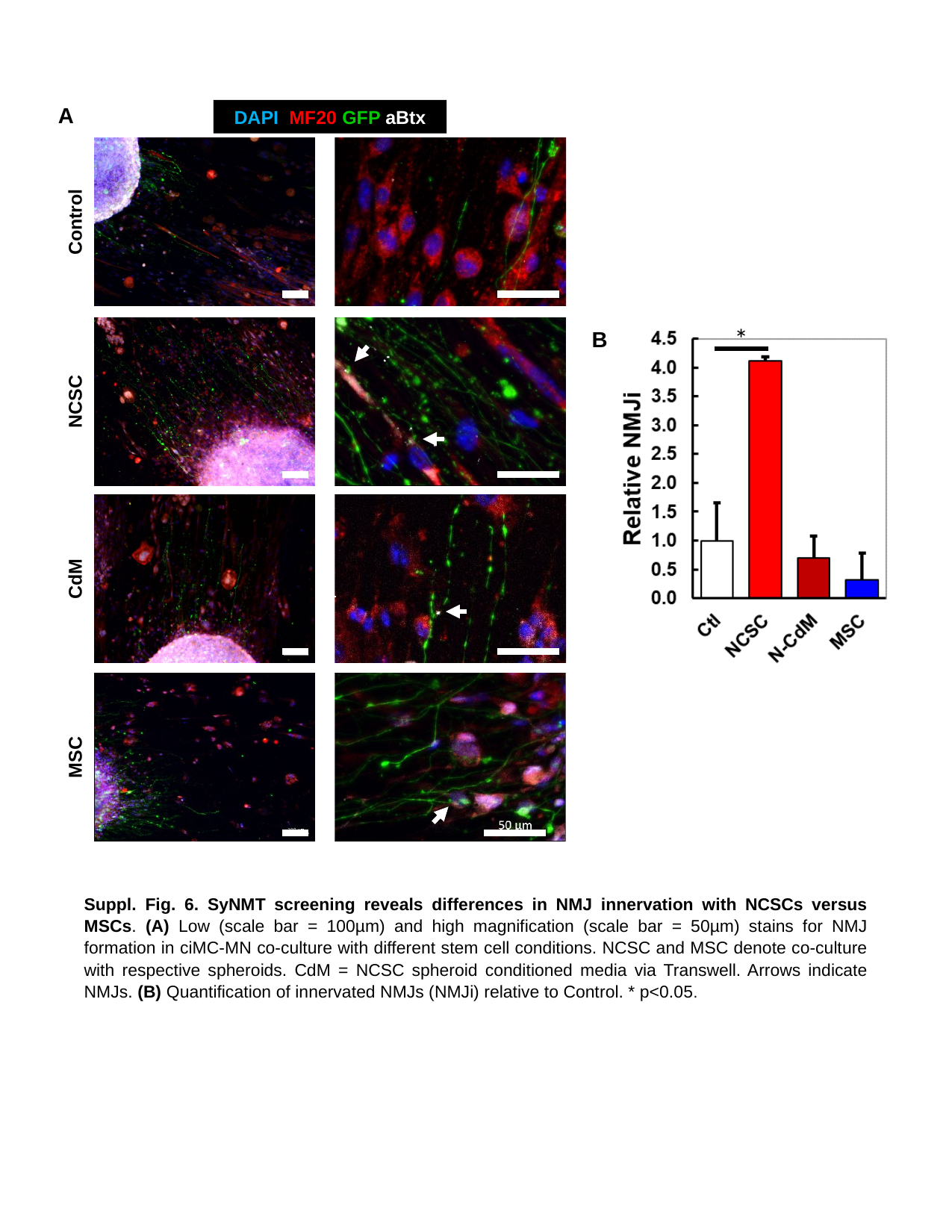

A
DAPI MF20 GFP aBtx
Control
*
B
NCSC
CdM
MSC
Suppl. Fig. 6. SyNMT screening reveals differences in NMJ innervation with NCSCs versus MSCs. (A) Low (scale bar = 100µm) and high magnification (scale bar = 50µm) stains for NMJ formation in ciMC-MN co-culture with different stem cell conditions. NCSC and MSC denote co-culture with respective spheroids. CdM = NCSC spheroid conditioned media via Transwell. Arrows indicate NMJs. (B) Quantification of innervated NMJs (NMJi) relative to Control. * p<0.05.

## Slide 7
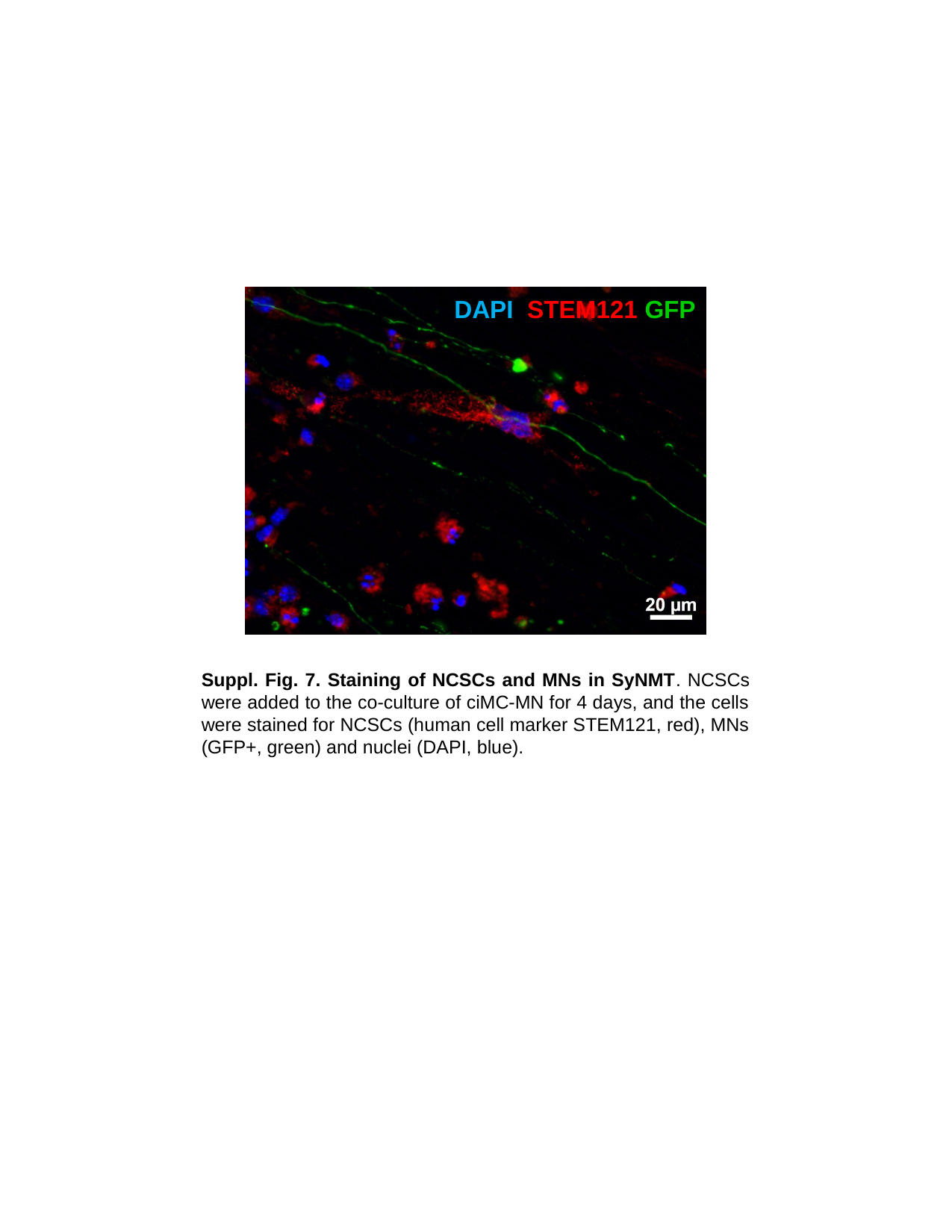

DAPI STEM121 GFP
Suppl. Fig. 7. Staining of NCSCs and MNs in SyNMT. NCSCs were added to the co-culture of ciMC-MN for 4 days, and the cells were stained for NCSCs (human cell marker STEM121, red), MNs (GFP+, green) and nuclei (DAPI, blue).
